# Emerging Role of E2F Family in Cancer Stem Cells

**DOI:** 10.3389/fonc.2021.723137

**Published:** 2021-08-12

**Authors:** Dan Xie, Qin Pei, Jingyuan Li, Xue Wan, Ting Ye

**Affiliations:** Department of Laboratory Medicine, The Affiliated Hospital of Southwest Medical University, Sichuan, China

**Keywords:** E2Fs, cancer stem cells, cancer, biomarkers, therapeutic targets

## Abstract

The E2F family of transcription factors (E2Fs) consist of eight genes in mammals. These genes encode ten proteins that are usually classified as transcriptional activators or transcriptional repressors. E2Fs are important for many cellular processes, from their canonical role in cell cycle regulation to other roles in angiogenesis, the DNA damage response and apoptosis. A growing body of evidence demonstrates that cancer stem cells (CSCs) are key players in tumor development, metastasis, drug resistance and recurrence. This review focuses on the role of E2Fs in CSCs and notes that many signals can regulate the activities of E2Fs, which in turn can transcriptionally regulate many different targets to contribute to various biological characteristics of CSCs, such as proliferation, self-renewal, metastasis, and drug resistance. Therefore, E2Fs may be promising biomarkers and therapeutic targets associated with CSCs pathologies. Finally, exploring therapeutic strategies for E2Fs may result in disruption of CSCs, which may prevent tumor growth, metastasis, and drug resistance.

## Introduction

**Figure 1:**
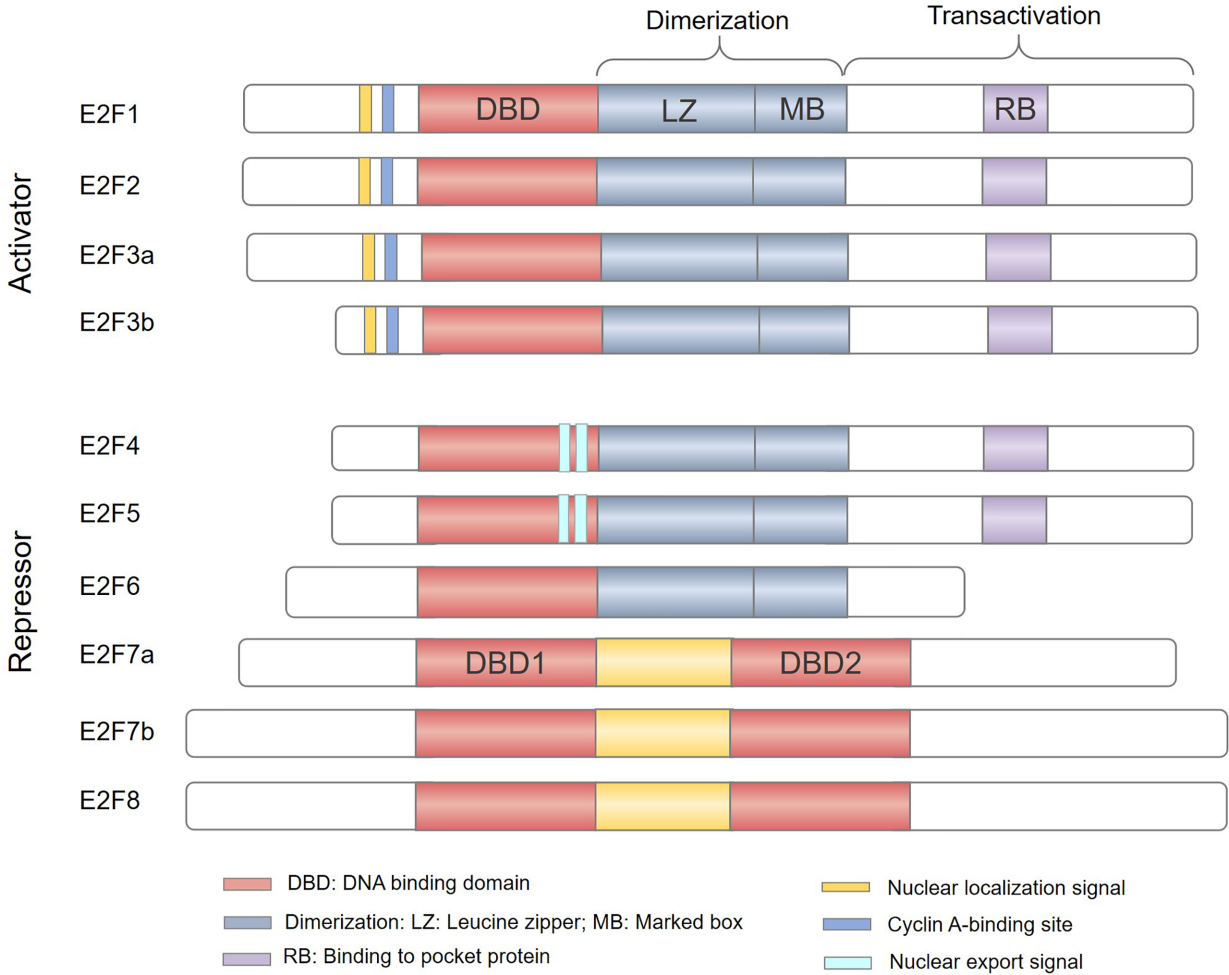
The E2F family of transcription factors. Based predominantly on the results of *in vitro* studies, E2Fs are generally divided into transcriptional activators (E2F1-3) and transcriptional repressors (E2F4-8). E2F family members E2F1–E2F6 contain a distinctive winged-helix DNA binding domain (DBD) and a dimerization partner (DP) binding domain composed of a leucine zipper (LZ) and a marker box (MB) domain to bind DNA. In addition, E2F1-5 has a pocket protein (RB, p107 and p130)-binding domain in the transactivation domain; the minimum site is shown in light purple (RB). E2F1-3 also have a nuclear localization sequence (NLS) and a Cyclin A-binding site, while E2F4-5 have a nuclear export signal (NES). E2F7-8 have two tandem DBDs but lack dimerization and transactivation domains and do not bind to DP or pocket proteins.

The study of the E2F family began in 1987 and E2Fs were initially identified as activators of the adenovirus gene E2 promoter that can induce host cell proliferation (1, 2). The E2F family of transcription factors (E2Fs) have been recognized for nearly 30 years. A total of 8 genes have been found, and 10 protein products encoded by these genes form a core transcription axis crucial for regulating cell cycle progression, apoptosis, differentiation, DNA damage repair, metabolism, and angiogenesis (3–7). According to initial reporter gene detection and evaluation of their expression patterns during the cell cycle, E2Fs have been classified as transcriptional activators (E2F1-3) or transcriptional repressors (E2F4-8) (**Figure 1**), and are thus predicted to play a dual role in human cancers (8, 9). E2F activators are predicted to be oncogenic, while E2F repressors are predicted to have tumor suppressor functions. However, due to the complexity of their structure and function, it is still quite challenging to study the role of E2Fs in cancer.

Cancer stem cells (CSCs, also called tumor-initiating cells) are a small subpopulation of cancer cells that possess self-renewal capacity and lead to the heterogeneous lineages of cancer cells that constitute the tumor, and they are largely responsible for tumor growth and progression (10). CSCs were first identified in acute myeloid leukemia (AML) and have since been purified from diverse types of solid malignancies, such as breast cancer (11), liver cancer (12), lung cancer (13), colon cancer (14), prostate cancer (15), ovarian cancer (16) and brain cancer (17). The CSC model provides a satisfactory explanation for the origin of complex tumors and intratumoral heterogeneity. Only a few CSCs with self-renewal and differentiation potential can initiate tumor formation and produce intratumoral heterogeneity (18). The CSC model has been well established for cancer research, and accumulated evidence has demonstrated that a high recurrence rate and high mortality rate in cancers are intimately related to the biological properties of CSCs (19, 20).

In cancer literature, the role of E2Fs in CSCs is widely regarded as an activator gene. Up-regulation of E2Fs is reported to be involved in proliferation promotion (21), maintenance and acquisition of self-renewal (22, 23), invasion and metastatic progression (24) and resistance to chemotherapy and radiotherapy (25) in many CSCs. However, the unexpected positive effect of repressive E2Fs on stem cell attributes of CSCs could potentially be a consequence of repression of negative regulators such as miRNAs (26). The discrepancies between these findings and those on E2Fs as a CSCs promoter or suppressor have not been thoroughly investigated. In addition, whether E2Fs may regulate the CSCs as it does in normal stem cells has not been clearly elucidated (27). In summary, this review has provided new evidence demonstrating the biological roles of E2Fs in CSCs and its underlying mechanisms, which opens up a new perspective for biomarkers or therapeutic targets for cancer.

## E2Fs: Structure And Functions

Currently, there are 8 E2F genes (called E2F1-8) that encode 10 proteins in mammalian cells (**Figure 1**). All proteins encoded by E2Fs contain one or more highly conserved DNA binding domains (DBDs), which regulate promoter expression by targeting transcription (28). Among E2F genes, the E2F3 and E2F7 loci can undergo two alternative splicing events to encode four protein isoforms: E2F3a, E2F3b, E2F7a, and E2F7b (29–31). The E2F family can be broadly divided into two categories, typical E2Fs (E2F1-6) and atypical E2Fs (E2F7-8), based on their unique structural characteristics (32, 33). Canonical E2F1-6 members possess a DBD upstream of a dimerization partner (DP) binding domain composed of a leucine zipper (LZ) and a marker box (MB) domain (33–35). Unlike E2F6, E2F1-5 have a transactivation domain in the C-terminus and contain a binding region for pocket proteins. Therefore, E2F1-5 are widely regulated by the pocket proteins (also called “retinoblastoma family proteins”) RB, p107 and p130 (36). In addition, E2F1-3 have a nuclear localization signal (NLS) and a cyclin A binding site in the N-terminus to ensure their translocation to the nucleus to regulate cell cycle activity (37, 38). E2F4-5 have bipartite nuclear export signals (NESs) that mediate their cytoplasmic translocation (39, 40). However, atypical E2F7 and E2F8 have two distinct DBDs but lack DP binding domains, pocket protein binding regions, and transcriptional activation domains (41, 42). Consequently, atypical E2F7-8 bind to DNA as homodimers or heterodimers *via* the DBD to regulate gene transcription (43). Owing to their structural differences (**Figure 1**), E2Fs are destined to perform different functions in the cells.

Based on their functional properties, E2Fs are generally subdivided into two categories: transcriptional activators (E2F1-3) and repressors (E2F4-8) (29). The transcriptional activity of E2F1-3a is dependent on cell cycle regulatory and their binding partners, which include DPs and pocket proteins. Pocket proteins (RB/p107/p130) exhibit varying degrees of binding specificity for E2F subunits. For example, E2F1-3a preferentially bind to RB (28, 44). Generally, when RB is hypophosphorylated, it can hinder the transcriptional activity of the E2F-DP heterodimer by masking the transcription activation domains of E2F1-3a (3, 35). During cell cycle progression from G1 to S phase, RB is phosphorylated by activated cyclin-dependent kinases (CDKs), resulting in its dissociation from the RB-E2F complex and the unmasking of the E2F1-3a transcriptional activation domain (45, 46). E2F3b acts as both a transcriptional activator and repressor protein due to its unique transcriptional program and expression patterns (47). E2F3b remains constitutively expressed throughout the cell cycle, similar to the E2F4-5 repressors (35, 46, 48). During cell cycle progression in G0 phase, E2F3b-5 interact with one of the three RB/p107/p130 proteins, subsequently recruiting corepressor complexes to alter the local chromatin structure of E2F target genes and inducing transcriptional repression (46, 49, 50). E2F6-8 are considered repressors that are independent of pocket proteins, and their primary function is to modulate cell cycle progression from S to G2 phase. E2F6 can suppress transcription by recruiting chromatin remodeling complexes (51), while E2F7-8 can directly modulate gene transcription (33, 52). E2Fs are critical regulators of the cell cycle, and they regulate every phase of the cell cycle by controlling the transcription of numerous target genes involved in DNA replication and cell cycle progression.

## CSCs: Biological Characteristics

Tumor initiation, development, metastasis, recurrence and acquisition of therapeutic resistance in numerous different human cancers has been attributed to the properties of CSCs, which include proliferation potential, self-renewal capacity, differentiation potential, high metastatic capacity, and drug resistance (20, 53). Compared with normal stem cells, CSCs, with their abnormal expression of cell cycle-related regulatory factors and dysregulation of negative feedback mechanisms, are often able to proliferate extensively, potentially indefinitely (54, 55).

A prominent feature of CSCs is their extraordinary self-renewal ability, a unique stem-cell associated cell division event maintaining the undifferentiated state and long-term proliferation potential of at least one daughter cell, which is the direct cause of tumorigenesis (10). CSCs can divide symmetrically producing two CSCs that are undifferentiated (amplification of renewing CSCs) or asymmetrically producing one undifferentiated CSC and one lineage-restricted and partially differentiated daughter cell excessively increases cell growth and eventually leads to together driving heterogeneous tumor formation (19, 56). Regardless of the degree of differentiation of a given tumor, the undifferentiated and self-renewing CSC subset provides for the long term proliferative potential driving tumor development, tumor maintenance and metastasis, and are thus widely considered the key link to tumorigenesis (57). Therefore, a further understanding the regulatory mechanism of CSC self-renewal is vital to preventing tumorigenesis, and it can also provide clear targets for cancer treatment.

Along with their self-renewal ability, CSCs also have differentiation potential. CSCs can differentiate into a series of distinct cell types present within the tumor, which constitute the bulk of the tumor (58). It should be noted, however, that CSC differentiation into non-stem tumor cells (non-CSCs) is not a one-way pathway but can be reversible or plastic (59). For example, tumor cells can also dedifferentiate and acquire stem cell properties in response to specific stimuli (56, 60). In summary, CSC populations are dynamic populations with high cellular plasticity. In heterogeneous tumor cell populations, cells can undergo phenotypic switching between CSCs and tumor cells phenotype, a phenomenon that is essential for tumor progression and recurrence (61, 62).

A high metastatic potential is another key trait of CSCs. *In vivo*, CSC populations in tumors preferentially metastasize, and single-cell analysis has shown that early metastatic cells have unique stemness gene expression patterns (63, 64). Epithelial‐to‐mesenchymal transition (EMT) is the basis for cell invasion and metastasis. It is currently clear that EMT signaling, which enhances the metastatic potential of CSCs, and CSC phenotypes are tightly connected (65, 66). These studies have shown that the metastatic potential of CSCs is far higher than that of ordinary tumor cells and plays a crucial role in tumor metastasis and development.

Furthermore, drug resistance is regarded as an important feature of CSCs. CSCs display high resistance to chemotherapy and radiotherapy. Many mechanisms have been proposed for CSC resistance, such as expression of multidrug resistance proteins, enhancement of the DNA repair capacity, inhibition of cell death-related pathways, apoptosis evasion, cell cycle promotion and metabolic alteration (67). Additionally, the hypoxia microenvironment is also a key component of CSC maintenance and acquisition of drug resistance, especially in the enhancement of drug resistance mechanisms (68, 69). Because of their therapeutic resistance, CSCs are considered to be the root of treatment failure and tumor recurrence.

**Figure 2:**
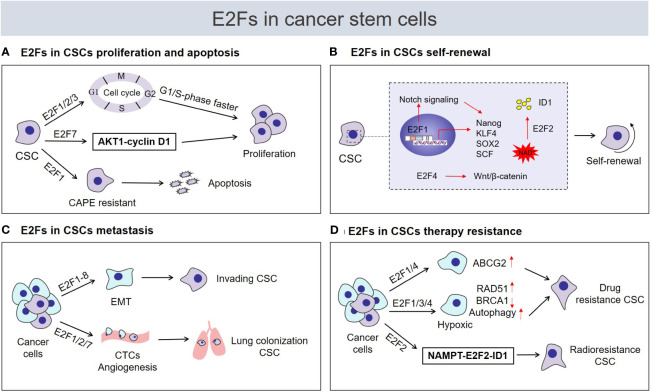
Roles of E2Fs in cancer stem cell (CSC) biological characteristics. Tumor cells are heterogeneous, including immortal cancer stem cells. CSCs usually express high levels of E2F transcription factors and are characterized by **(A)** enhanced proliferation or induction of apoptosis, **(B)** acquisition and maintenance of self‐renewal capability, **(C)** enhanced invasion and metastasis potential, **(D)** regulation of drug or radiation resistance. CAPE, caffeic acid phenethyl ester; ID1, inhibitor of differentiation 1; NAD^+^, nicotinamide adenine dinucleotide; CTCs, circulating tumor cells; NAMPT, nicotinamide phosphoribosyl transferase.

A continually increasing number of studies have researched E2Fs in CSCs and have found that E2Fs are widely involved in the regulation of the biological characteristics of CSCs, such as their proliferation, self-renewal, metastasis, and drug resistance **(** **Figure 2** **)**.

## Role Of E2Fs In The Biological Behaviors Of Cancer Stem Cells

### E2Fs Roles in Proliferation and Apoptosis of Cancer Stem Cells

E2Fs are critical regulators of genes required for cell cycle progression and play an integral role in the control of cell proliferation. Previous studies have suggested that E2Fs are required to control cell proliferation differently in carcinogenic environments than in normal cells (29). As shown in recent studies, the proliferative potential of CSCs seems to be strongly correlated with cell cycle regulation by E2Fs. The transcriptional activators E2F1-3 regulate cell proliferation by activating genes essential for G1/S-phase progression in CSCs **(** **Figure 2A** **)**. ENPP1 (ectonucleotide pyrophosphatase/phosphodiesterase 1) has been proved to induce the stemness features of cancer cells (70, 71). Bageritz et al (72) also discovered that E-NPP1 could facilitate glioblastoma CSC proliferation by controlling cell cycle progression. And E-NPP1-deficient glioblastoma CSCs led to a decrease in the transcriptional function of E2F1, and classical E2F1 target genes facilitating G1/S transition were downregulated, resulting in the accumulation of cells in G1 phase and inhibition of their proliferation. Similarly, E2F1 plays a pivotal role in regulating the proliferation status of chronic myeloid leukemia (CML) stem/progenitor cells (SPCs). In terms of cell cycle regulation, the E2F1 signaling pathway was found to be deregulated in CML SPCs, and inhibition of E2F1 led to cell cycle arrest and induced blockade of proliferation (21). Moreover, the dissociation of E2F1 from the RB protein was found to restore its transcriptional activity, which is required for cell cycle progression from G1 to S phase (73). In glioblastoma CSCs, the bromodomain inhibitor JQ1 was found to cause cell cycle arrest and suppress CSC proliferation by preventing the release of E2F1 from the RB/E2F1 complex (74). Additionally, glioblastoma CSCs display the dependence of HELLS (helicase, lymphoid-specific) to maintain proliferation. Both E2F3a and E2F3b were found to interact with HELLS, thereby increasing the expression of cell cycle progression-related genes to maintain glioblastoma CSC proliferation, while proliferation was impaired after E2F3 gene knockdown (75). Remarkably, although the function of E2F repressors is generally opposite that of E2F activators in the regulation of the cell cycle, the effects of E2F repressors are inconsistent with the predicted inhibitory effect on proliferation. In fact, current experimental evidence supports the role of E2F repressors in promoting cell cycle progression and proliferation of CSCs. For example, high levels of E2F7 detected in liver CSC populations were found to be vital for the maintenance of CSC proliferation *via* E2F7 activation of AKT1-cyclin D1 signaling and downstream cell cycle mediators to promote cell cycle progression and proliferation (26) **(** **Figure 2A** **)**. The unexpected positive effect of repressive E2Fs on stem cell attributes of CSCs, such as proliferation by modulation of the cell cycle could potentially be a consequence of repression of negative regulators such as miRNAs (see below). However, E2F1 is also an effective activator of apoptosis (34), which may lead to the opposite effect of E2F1 on proliferation of CSCs. As demonstrated by El-Khattouti et al (76), the apoptosis of CD133 (+) melanoma subpopulation cells induced by the anticancer drug caffeic acid phenethyl ester (CAPE) requires the participation of E2F1. Functional analysis of E2F1 in the CD133(+) melanoma subpopulation demonstrated that overexpression of E2F1 induced both degradation of the antiapoptotic protein Mcl-1 and activation of the ASK1/JNK and p38 pathways, which are involved in regulating the localization of Bax to mitochondria to trigger apoptosis (76) **(** **Figure 2A** **)**. Overall, E2Fs mainly regulate the proliferation of CSCs in a cell cycle-dependent manner, and E2Fs are seem to necessary factors in the control of CSC proliferation. But E2F1 can promote the apoptosis of CSCs, whether this is attributed to the CSC-type specific or other mechanisms is worth further study.

### E2Fs in the Maintenance and Acquisition of Cancer Stem Cell Self-Renewal

Self-renewal is the process by which cells divide while maintaining an undifferentiated state. This process requires maintenance of stem cell transcription factor expression, activation of self-renewal signaling pathways and strict control of cell metabolism (77–79). Accumulating evidence suggests the substantial engagement of distinct E2F members in each of the abovementioned self‐renewal programs and the contributions of E2Fs to stemness acquisition and maintenance of the self-renewal ability of CSCs. Several studies have confirmed that E2F1 can directly transcriptionally activate the stem cell transcription factors NANOG, KLF4, SOX2 and SCF to act as a regulator of CSC self‐renewal **(** **Figure 2B** **)**. For example, using liver tumor-initiating stem-like cells (TICs), Chen et al (80) demonstrated that E2F1 transcriptionally activated NANOG expression, which in turn inhibited oxidative phosphorylation (OXPHOS) and activated fatty acid oxidation (FAO) through metabolic reprogramming, maintaining the TIC self-renewal ability. In ovarian CSCs, E2F1 acts as an upstream transcription factor to activate KLF4 expression and participates in EIF5A2-mediated modulation of CSC self-renewal properties (81). In addition, YAP1 is known to be a major mediator of Hippo signaling pathway and plays an important role in CSC self-renewal (82). Schaal et al (83) proved that E2F1 transcriptionally activates the Sox2 promoter *via* YAP1 mediated in response to the signaling events caused by the binding of nicotine to α7 nAChR, which enhances the self-renewal of lung CSCs. In the same context, E2F1-3a was also found to induce SCF expression at the transcriptional level under nicotine exposure. Research on the underlying mechanism has mainly involved E2F1. When the binding of nicotine to nAChRs initiates a signaling cascade, it results in the induction of E2F1-mediated SCF promoter activation and stimulates c-Kit expressed on stem-like cells, facilitating the self-renewal of lung cancer side population cells (84). Additionally, the NOTCH and WNT/β-catenin signaling pathways are essential for the self-renewal of CSCs (57). Limited studies have demonstrated regulation of the NOTCH and β‐catenin pathways by E2Fs to promote the acquisition of self-renewal capability. In prostate cancer cells, this involvement of MUC1-C (mucin 1 C-terminal transmembrane subunit) in lineage plasticity is associated with induction the CSCs state and self-renewal capacity (85, 86). A recent study confirmed that MUC1-C could directly bind to E2F1 activated the BAF remodeling complex, induced NOTCH1 signaling pathway and promoted NANOG expression, which in turn led to the promotion of CSCs stemness (86). An additional study reported that TREX1 (three prime repair exonuclease 1) negatively regulates the self-renewal of osteosarcoma CSCs. With respect to underlying mechanisms, TREX1 suppression allowed the activation of β-catenin signaling in the dependence of E2F4, thus possibly enhancing the self-renewal ability of osteosarcoma CSCs by upregulating OCT4 (87) **(** **Figure 2B** **)**. Intriguingly, a recent finding suggests crosstalk among E2F2, the NAD^+^ biosynthesis pathway and CSC self‐renewal. Gujar et al (25) found that NAD^+^ metabolism governs glioblastoma CSC self-renewal *via* the NAMPT-E2F2-ID axis. NAMPT is the rate-limiting enzyme in the NAD salvage pathway, and E2F2 acts downstream of NAMPT and controls ID1 gene transcription to drive glioblastoma CSC self-renewal. E2F2 is required for the link NAD^+^ metabolism and the self-renewal transcriptional program in glioblastoma CSCs **(** **Figure 2B** **)**.

Recent studies have highlighted that E2Fs promote CSC self‐renewal by enhancing the activity of stem cell transcription factors, regulating self‐renewal signaling pathways, and modulating cell metabolism. However, to unequivocally define E2Fs as regulators of CSC self‐renewal, further studies are needed to clarify that E2Fs are required for the regulation of CSC self‐renewal.

### The Roles of E2Fs in Cancer Stem Cell Metastasis

Metastasis is the process by which malignant tumor cells travel from the primary site through lymphatic vessels, blood vessels, or body cavities and spread to other sites (88). In metastasis, CSCs show more invasive and metastatic phenotypes than non-stem tumor cells and play the lead role (89). Because CSCs have the ability to initiate tumors and a high metastatic capacity, they are considered to be the cells responsible for repopulating metastatic tumor (89, 90). In recent years, emerging research evidence has documented that some circulating tumor cells (CTCs) are CSC-like cells with strong metastatic potential that can drive macroscopic tumor growth in distant tissues (91–95). Interestingly, in a mouse model of metastatic breast cancer, the lung colonization capacity of CTCs and the numbers of metastatic lesions in the lung were strikingly reduced in mice on both the E2F1 and E2F2 knockout backgrounds (24, 96) **(** **Figure 2C** **)**. Moreover, E2F1 loss also caused loss of VEGFA expression and tumor angiogenesis defects (24). CSCs are mostly located around blood vessels and rely on the microvasculature for metastasis (97, 98). These findings raise the possibility that cell invasiveness and tumor angiogenesis regulated by the activity of E2F1/2 might be effectively involved in the metastasis of CSC-like cells, thus facilitating tumor metastasis. Moreover, a recent study by Teng et al. (99) found that upregulation of E2F7 resulted in increased activation of the VEGFR-2 signaling pathway and promoted metastasis, invasion, and angiogenesis in hepatocellular carcinoma (HCC). Concomitantly, high expression of E2F7 in HCC stem cells (26) provides the potential for E2F7 to enhance CSC metastasis.

EMT is closely related to the migration and invasion of CSCs. Of note, EMT combines two of the most important attributes to facilitate metastasis: invasiveness and stemness (89, 100). To the best of our knowledge, E2F1-8 extensively activate the EMT program in a variety of cancers, leading to the acquisition of a very invasive and metastatic phenotype (101–106), which may further result in the acquisition of a metastatic CSC phenotype **(** **Figure 2C** **)**.

To date, the exact mechanism of several E2Fs in the regulation of CSC metastasis is still unclear, although their involvement seems essential. Further research is necessary to understand how E2F-dependent regulation of EMT and angiogenesis are interconnected in CSC metastasis.

### E2Fs Regulate the Drug Resistance of Cancer Stem Cells

CSCs are the main culprit of resistance to chemotherapy and radiotherapy. The mechanisms underlying drug resistance involves in efficiently inducing drug efflux through ATP-binding cassette (ABC) transporters (including MDR1, MRP1, and ABCG2), activating DNA repair programs, enhancing autophagy, and/or inhibiting apoptotic signals to avoid death (67, 107). In a variety of human cancers, the resistance mechanism of ABCG2 protein overexpression in CSCs has been used as an effective functional mechanism to identify and isolate CSCs (19). E2F1 was further reported to drive chemotherapeutic drug resistance in lung and breast cancer cells *via* activation of ABCG2 expression (108). This observation demonstrates new scenarios that E2F1 may participate in ABCG2-mediated regulation of CSC resistance. Additionally, an increasing amount of evidence shows that tumor cells under hypoxic conditions are induced to transform into CSCs phenotype and mediate tumor radiotherapy or chemotherapy resistance (68). Meanwhile, many cancer literatures have investigated the close link between E2Fs and hypoxia. For example, E2F1 can transactivate the RAD51 promoter under hypoxia to facilitate homologous recombination repair and drug resistance of prostate cancer cells (109). E2F4 was reported to repress BRCA1 expression with hypoxia-induced (110). Concomitantly, DNA repair gene BRCA1 is a critical tumor suppressor that helps CSCs acquire drug resistance under hypoxic conditions (111). And E2F1 and E2F3 can mediate autophagy activation of cancer cells with hypoxia (112, 113). Moreover, autophagy caused by hypoxia commonly results in enhancing CSCs chemoresistance (114, 115). Thus, this set of observations indicate the possibilities that E2Fs could induce the DNA repair and autophagy enhancement through hypoxia regulation, thereby increasing CSC drug resistance. Other studies have also suggested that E2F2 mediates the radioresistance of glioblastoma CSCs (25). The NAMPT-E2F2-ID1 pathway was found to be upregulated in glioblastoma CSCs after radiation and represented a protective response to radiation **(** **Figure 2D** **)**.

In summary, these studies, together with the limited research focus on E2Fs and CSC resistance, accentuate the necessity to redirect our attention to the study of E2Fs, because these proteins perform unique transcriptional functions to mediate resistance mechanisms in CSCs.

### E2F-Related miRNAs in Regulation of Cancer Stem Cells

MicroRNAs (miRNAs) are small noncoding RNAs that function as posttranscriptional regulators. Previous works have described relationships between the expression of miRNAs and E2Fs. E2F activity can be regulated by miRNAs; in contrast, miRNAs themselves are targets of E2F proteins (116). Subsequent studies found that this feedback relationship plays a pivotal role in CSCs **(** **Table 1** **)**. Regarding E2F1, oncogenic miR-20b-5p was found to increase the proliferation of breast CSCs by upregulating E2F1 protein expression (117). Concomitantly, E2F1 was found to act as a downstream target of miR-185-3p and promote CSC stemness properties through the LINC00511/miR-185-3p/E2F1/Nanog axis in breast cancer (118). Additionally, regulation of the E2F1 pathway by miRNAs is a novel strategy for combating CSCs. For example, hsa-mir183/EGR1–mediated upregulation of E2F1 is required for CML SPC survival and proliferation. Downstream of this event, inhibition of E2F1 was found to reduce the proliferation of CML SPCs and induce p53-mediated apoptosis, but healthy SPCs were not affected by inhibition of the E2F1 pathway (21). The researches above indicated that E2F1 seems to be a positive regulator of CSC-traits. However, E2F1 can also negatively regulate the characteristics of CSC. In gastric CSCs, upregulated miRNA-20a was found to contribute to the self-renewal and proliferation of CSCs by targeting the inhibition of E2F1 protein expression and subsequently activating Wnt/β-catenin signaling (119, 120). Taken together, E2F1-mediated regulation promotes the properties of breast cancer and CML CSCs, but has an inhibitory effect on the characteristics of gastric CSCs. And targeting blockage of E2F1 anticipated is expected to be a potential therapeutic applicable to breast cancer and CML. However, whether the promotion and inhibition of E2F1-mediated CSCs regulatory activities are tumor-type specific or miRNAs regulation, which requires further evidences to predict outcome.

**Table 1:** Roles of E2F-related miRNAs in regulation of cancer stem cells.

Cancer stem cell type	E2F with upregulated expression	Related miRNAs	Effects on CSC properties		Ref. No.
			Induction	Suppression	
Breast CSCs	E2F1	miR-20b-5p	Proliferation		(117, 118)
		miR-185-3p	Stemness		
Gastric CSCs	E2F1	miR-20a		Self-renewal Proliferation	(119, 120)
CML SPCs	E2F1	miR-183	Proliferation Survival		(21)
Glioblastoma CSCs	E2F2	miR‐125b	Proliferation		(121, 122)
		Let-7b			
TNBC CSCs	E2F2	miR-4319	Self-renewal Metastasis		(123)
Breast CSCs	E2F2	miR-638	Self-renewal		(124)
			Proliferation		
			Invasion		
Lung CSCs	E2F2	miR-99	EMT		(125)
			Stemness		
Colon CSCs	E2F3	miR-449b	Proliferation		(126)
Glioblastoma CSCs	E2F3	miR128-1	Proliferation		(127)
Ovarian CSCs	E2F6	miR‐193a	Stemness		(22, 128)
Liver CSCs	E2F7	miR-302a/d	Self-renewal		(26)
			Proliferation		
Colon CSCs	E2F7	miR-199b	Stemness		(23)
			5-FU resistance		

A variety of miRNAs regulate the biological characteristics of CSCs by directly targeting E2F2 **(** **Table 1** **)**. For instance, miR‐125b was found to suppress the proliferation of CD133-positive glioblastoma CSCs by direct downregulation of E2F2 (121). Elevated Let-7b also repressed E2F2 expression in glioblastoma CSCs, resulting in reductions in tumorsphere growth and CSC populations (122). In triple-negative breast cancer (TNBC), miR-4319 was found to negatively regulate the self-renewal and metastasis of CSCs through targeted inhibition of E2F2 (123). Concomitantly, overexpression of miR-638 also inhibited the self-renewal, proliferation, and invasion abilities of breast CSCs by suppressing E2F2 (124). Moreover, E2F2 expression was shown to inversely correlate with miR-99 expression and directly with elevated level of vimentin in lung cancer biopsies. By inhibiting E2F2, miR-99a repressed the EMT process, accompanied by suppression of stemness features, consequently decreasing the CSC population (125). Collectively, targeting E2F2 is expected to become a therapeutic target for a variety of CSCs.

Fewer studies have been devoted to studying the role of miRNAs related to the remaining E2Fs (E2F3-E2F8) in CSCs **(** **Table 1** **)**. For example, miR-449b was found to inhibit the proliferation of SW1116 colon CSCs through downregulation of E2F3 expression (126). miR128-1 was found to suppress the proliferation and tumorigenicity of glioblastoma CSCs by targeting E2F3 (127). Furthermore, E2F7 was found to be the direct target of miRNA-302a/d. miRNA-302a/d negatively regulates the self-renewal ability and cell cycle entry pathway of liver CSCs through direct repression of the target gene E2F7 and its downstream AKT/β-catenin/CCND1 signaling pathway (26).

On the other hand, E2Fs play a role in CSCs by regulating the transcription and maturation of various miRNAs **(** **Table 1** **)**. Recent research findings have suggested that E2F6 functions as a competing endogenous RNA (ceRNA) for miR‐193a. Upregulation of E2F6 mRNA expression, in turn, was found to upregulate the stemness marker c-Kit through E2F6-mediated silencing of miR‐193a and to promote ovarian cancer stemness and tumorigenesis (22, 128). In colon CSCs, E2F7 was found to transcriptionally inhibit miR-199b expression to promote USP47 expression, thereby increasing colon CSC stemness and accelerating the occurrence of colon cancer (23). In addition, E2F7 silencing was shown to decrease the production of ALDH1^+^ cells and repress antagonistic effects of ALDH1^+^ cells on 5-fluorouracil (5-FU) treatment.

The above findings show that E2Fs and miRNAs regulate one another and play important roles in the proliferation, self-renewal, metastasis, and drug resistance of CSCs. However, there are still some E2Fs (E2F4, E2F5 and E2F8) that are relatively infrequently studied in CSCs, and the related miRNAs have not been studied, which may be the direction of future research.

## E2Fs Are Novel Biomarkers And Therapeutic Targets Associated With CSCs

In recent studies, according to published bioinformatics papers, E2Fs have been found to be dysregulated in most tumors and can be considered prognostic and diagnostic biomarkers for cancer as well as potential therapeutic targets (129–136). As demonstrated previously, E2Fs are widely involved in the regulation of the biological characteristics of CSCs and play an important role in the occurrence, progression, metastasis and drug resistance of cancer, suggesting that they might be novel cancer biomarkers and therapeutic targets for CSC-associated. Indeed, using the logic model of core circuits, Khan et al (102) identified several EMT receptor proteins that, in combination with E2F1 upregulation, could be regarded as more reliable biomarkers for predicting the malignant progression of bladder cancer and breast cancer. In invasive bladder cancer and breast cancer, the survival times of patients with low expression of E2F1 and EMT receptor protein signatures were found to be approximately twice as long as those of patients with high expression (102). Similarly, Lee et al. (137) found that high expression of the E2F1-EZH2-SUZ12 signature reflected the invasion and CSC-like characteristics of bladder cancer and predicted poor prognosis of patients. In CD133(+) cells isolated from human astrocytomas, the expression of E2F2 was found to be upregulated and associated with the transformation of human astrocytes. Therefore, it was suggested that E2F2 be used as a therapeutic target for astrocytoma eradication (138). In high-grade muscle-invasive bladder cancer (MIBC), E2F3 is often highly expressed in the 6p22 amplification region, similar to the stem-like signature. E2F3 is epigenetically regulated and might be a potential therapeutic target (139, 140). In addition, E2F7 is highly expressed in HCC and colon cancer and can promote stemness in these cancers, suggesting that E2F7 may be a novel therapeutic target for HCC and colon cancer (23, 26). There are few studies on other E2F family members (E2F4, E2F5, E2F6, E2F8) in CSCs, but these E2Fs also have diagnostic and prognostic value and are therapeutic targets in different cancers. The E2F4 activity level can be used as an indicator of the survival and prognosis of patients with breast cancer and prostate cancer. Patients with negative E2F4 activity scores tend to survive, while upregulation of E2F4 activity is related to poor prognosis (141, 142). The E2F5 status significantly improves the diagnostic accuracy in epithelial ovarian cancer (OEC), and the presence of CA125 or E2F5 increases the sensitivity of OEC detection to 97.9% and the specificity to 72.5% (143). Additionally, E2F5 was identified as an independent prognostic factor in esophageal squamous cell carcinoma; the 5-year survival rate of the E2F5-positive group was 39.3%, which was significantly lower than that of the E2F5-negative group (83.8%) (144). In glioblastoma, research by Huang et al (145) showed that E2F6 is a potential therapeutic target for combating temozolomide (TMZ) resistance and that the progression-free survival (PFS) times of TMZ-treated patients with high levels of E2F6 were significantly shorter. Finally, E2F8 was identified as a novel therapeutic target for controlling the progression of lung cancer and liver cancer (43, 146). High E2F8 expression was found to be associated with poor RFS in patients with ER+ breast cancer (147). The above studies showed that E2Fs, either alone or in combination with other proteins or markers, can be used as diagnostic and prognostic biomarkers or therapeutic targets for cancer in a tumor-type specific manner.

## Conclusions And Future Perspectives

In summary, E2Fs are major players in regulating the biological characteristics of CSCs. E2Fs regulate the proliferation, self-renewal, metastasis, and drug resistance of CSCs *via* distinct mechanisms and can be considered stemness regulators in tumors. Therefore, E2Fs play integral roles in tumor growth, progression, metastasis and anticancer drug resistance. Notably, E2Fs are highly expressed in most solid tumors and are closely related to malignant progression and poor prognosis. A large number of studies have suggested that E2Fs can be used as new diagnostic and prognostic biomarkers and are potential therapeutic targets for cancer. However, there are still some problems that need to be solved. For example, do E2F transcriptional repressors promote the proliferation of CSCs? What are the regulation roles of E2Fs involved in CSCs apoptosis? How do E2Fs regulate EMT and tumor angiogenesis to affect CSC metastasis? Most importantly, there is no specific drug targeting E2Fs, a limitation that needs further exploration. Future research focused on answering the above questions may help us to better understand the roles of E2Fs in CSCs and may be a key step in combating CSCs.

## Author Contributions

DX and QP contributed to drafting and editing the manuscript, shared the first authorship. JL and XW contributed to the literature search. TY participated in the design, revision and finalization of the manuscript. All authors contributed to the article and approved the submitted version.

## Funding

This work was supported by the National Natural Science Youth Fund, China (Grant No. 82003138), the Sichuan Science and Technology Program for key Research and Development, China (Grant No. 2021YFS0226), Doctoral Research Initiation Fund of Affiliated Hospital of Southwest Medical University, China (Grant No. 19077) and University’s scientific research project of Southwest Medical University, China (Grant No. 2020ZRQNA019).

## Conflict of Interest

The authors declare that the research was conducted in the absence of any commercial or financial relationships that could be construed as a potential conflict of interest.

## Publisher’s Note

All claims expressed in this article are solely those of the authors and do not necessarily represent those of their affiliated organizations, or those of the publisher, the editors and the reviewers. Any product that may be evaluated in this article, or claim that may be made by its manufacturer, is not guaranteed or endorsed by the publisher.
